# Epigenetic mechanism of RBM15 in affecting cisplatin resistance in laryngeal carcinoma cells by regulating ferroptosis

**DOI:** 10.1186/s13062-024-00499-6

**Published:** 2024-07-23

**Authors:** Yue Liang, Haoyue Zhong, Yi Zhao, XiaoMin Tang, Chunchen Pan, Jingwu Sun, Jiaqiang Sun

**Affiliations:** Department of Otorhinolaryngology-Head and Neck Surgery, The First Affiliated Hospital of USTC, Division of Life Sciences and Medicine, University of Science and Technology of China, 17 Lujiang Road, Luyang District, Hefei, 230001 Anhui China

**Keywords:** Laryngeal carcinoma, Cisplatin resistance, RBM15, KDM5B, Ferroptosis, LncRNA FER1L4, LncRNA KCNQ1OT1, GPX4, ACSL4

## Abstract

Laryngeal carcinoma (LC) is a common cancer of the respiratory tract. This study aims to investigate the role of RNA-binding motif protein 15 (RBM15) in the cisplatin (DDP) resistance of LC cells. LC-DDP-resistant cells were constructed. RBM15, lysine-specific demethylase 5B (KDM5B), lncRNA Fer-1 like family member 4 (FER1L4), lncRNA KCNQ1 overlapping transcript 1 (KCNQ1OT1), glutathione peroxidase 4 (GPX4), and Acyl-CoA synthetase long-chain family (ACSL4) was examined. Cell viability, IC_50_, and proliferation were assessed after RBM15 downregulation. The enrichment of insulin-like growth factor 2 mRNA-binding protein 3 (IGF2BP3) and N6-methyladenosine (m6A) on KDM5B was analyzed. KDM5B mRNA stability was measured after actinomycin D treatment. A tumor xenograft assay was conducted to verify the role of RBM15 in LC. Results showed that RBM15 was upregulated in LC and its knockdown decreased IC_50_, cell viability, proliferation, glutathione, and upregulated iron ion content, ROS, malondialdehyde, ACSL4, and ferroptosis. Mechanistically, RBM15 improved KDM5B stability in an IGF2BP3-dependent manner, resulting in FER1L4 downregulation and GPX4 upregulation. KDM5B increased KCNQ1OT1 and inhibited ACSL4. KDM5B/KCNQ1OT1 overexpression or FER1L4 knockdown promoted DDP resistance in LC by inhibiting ferroptosis. In conclusion, RBM15 promoted KDM5B expression, and KDM5B upregulation inhibited ferroptosis and promoted DDP resistance in LC by downregulating FER1L4 and upregulating GPX4, as well as by upregulating KCNQ1OT1 and inhibiting ACSL4. Silencing RBM15 inhibited tumor growth in vivo.

**Supplementary Information:**

The online version contains supplementary material available at 10.1186/s13062-024-00499-6.

## Introduction

Laryngeal carcinoma (LC) is a malignant tumor that arises from the epithelial cells of the laryngeal mucosa and usually manifests as tumors of the vocal cords, glottis, and ventricles of the larynx [1]. Laryngeal cancer (LC) ranks as one of the most prevalent head and neck cancers, with approximately 170,000 new cases and 90,000 fatalities annually. Moreover, the survival rate for advanced-stage patients is below 50% [2]. Clinical manifestations of LC include neck masses, difficulty swallowing, hoarse voice or airway obstruction, persistent hoarseness, ear pain, difficulty in swallowing, chronic cough, wheezing, and hemoptysis [3]. Depending on the tumor node metastasis stage and the primary site of LC, various combinations of surgery, radiotherapy, and chemotherapy are usually used for treatment, but these treatments may cause organ dysfunction [4]. Cisplatin (DDP), a platinum compound widely used in cancer therapy, inhibits tumor cell proliferation and survival by binding to DNA and interfering with DNA replication and transcription processes [5]. DDP has been widely used for LC treatment and has shown significant therapeutic effects, but the long-term application of DDP therapy often leads to drug resistance, which becomes one of the major challenges in the treatment of LC [6]. Ferroptosis represents a recently identified mechanism of cell death, marked by an upsurge in intracellular free iron and an overabundance of reactive oxygen species (ROS), leading to the cell membrane rupture and oxidative damage of important molecules in the cytoplasm [7, 8]. Given that cancer cells consume more iron than normal cells and that resistant cancer cells are sensitive to ferroptosis, ferroptosis is not only an excellent option to trigger cell death and reverse resistance but also provides therapeutic selectivity [9, 10]. However, much remains unknown about the molecular mechanisms and interactions between ferroptosis and DDP resistance in LC, which will need to be revealed by further studies.

6-methyladenosine (m6A) is the most prevalent internal cotranscriptional modification in eukaryotic RNA, which is modified by m6A methyltransferases (writers), removed by demethylases (erasers), and recognized by m6a binding proteins (readers) [11]. RNA binding motif (RBM) proteins are a class of RNA binding proteins involved in RNA metabolism, including splicing, transport, and translation [12]. As a member of RBM proteins, RBM15 is a methyltransferase that can direct methyltransferase proteins to specific RNA sites for m6A modification and regulate long non-coding RNA (lncRNA) [13]. Interestingly, RBM15 is highly expressed in LC, accompanied by upregulation of mRNA m6A methylation through an IGF2BP3-dependent manner [14]. In addition, a previous study has confirmed that RBM15 has an impact effect on chemoresistance through mRNA methylation in ovarian cancer [15]. However, the effect of RBM15 on DDP resistance through m6A modification in LC is still unclear.

Lysine-specific demethylase (KDM) dynamically regulates histone methylation, demethylating histone H3 lysine 4 (H3K4), H3К9, H3K27, and H3K36 through xanthine-adenine dinucleotide, Fe^2+^, and α-ketoglutarate, thus playing an important role in regulating chromatin dynamics and transcription [16]. KDM5B, a member of the KDM family, catalyzes H3K4me2/3 demethylation through transcriptional repression and is involved in DNA repair, drug resistance, and cancer immunotherapy [17]. Of note, the KDM5B mRNA stability can be regulated in an m6A-dependent manner [18]. Another report indicated that alkylation repair homolog protein 5-mediated m6A demethylation reduced H3K4me3 methylation by altering the expression of KDM5B [19]. KDM5B is considered an oncogene that promotes the proliferation and invasion of many malignant tumors [20–22]. More importantly, KDM5B is highly expressed in LC and associated with poor prognosis, lymphatic metastasis, and recurrence, which may be achieved through the regulation of H3K4me3 modification [23]. Similarly, the promotion of malignant biological behaviors in LC by KDM5B may be achieved by promoting SRY-box (SOX) transcription factor 2 expression through H3K27me3 demethylation [24]. KDM5B demethylates H3K4me3 on the SOX17 promoter, thereby inhibiting SOX17 expression [25]. Based on the above evidence, we aim to further investigate the mechanism of m6A modification in stabilizing KDM5B mRNA level and the role of KDM5B demethylation of H3K4me3 and H3K27me3 in LC.

The long non-coding RNA FER1L4, spanning 6.7 kilobases and situated at 20q11.22 [26], has been associated with tumor proliferation, invasion, migration, and apoptosis in various cancers, including gliomas [27], oral squamous cell carcinoma [28], and liver cancer [29]. In cancer tissues such as breast cancer, enriched H3K4me3 and H3K27ac at the FER1L4 promoter were observed, while H3K27me3 was less prevalent at the FER1L4 promoter [30]. More importantly, FER1L4 was significantly down-regulated in LC, and the up-regulation of FER1L4 inhibited the invasion and migration of LC cells [31]. KCNQ1 overlapping transcript 1 (KCNQ1OT1) is a 91 kb unspliced lncRNA located on chromosome 11p15.5 and implicated in cell proliferation, cell cycle, metastasis, and immune evasion in cancer [32]. Gene silencing by KCNQ1OT1 RNA involves repressive histone modifications, including H3K27me3 [33]. In our investigation, RNAInter database analysis indicated potential binding between FER1L4 or KCNQ1OT1 and the transcription factor myelocytomatosis oncogene (MYC), while MYC was found to potentially bind to ferroptosis-related proteins glutathione peroxidase 4 (GPX4) or Acyl-CoA synthetase long-chain family member 4 (ACSL4). As a result, we postulated that FER1L4 might bind to the transcription factor MYC to suppress the expression of GPX4, and KCNQ1OT1 might bind to MYC to suppress the expression of ACSL4, consequently impeding ferroptosis and leading to DDP resistance in LC cells.

In this study, we established a DDP-resistant LC cell line and investigated the role of RBM15 in the drug resistance in LC cells, aiming to find new targets for improving the effect of chemotherapy on LC.

## Materials and methods

### Cell culture

LC cell lines (AMC-HN-8, TU686, HNO210, TU177) and normal bronchial epithelial cell lines 16HBE (ATCC, Manassas, VA, USA) were cultured in Dulbecco’s modified Eagle’s medium (DMEM, Gibco, Grand Island, NY, USA) containing 100 unit/mL penicillin, 100 ng/mL streptomycin, and 10% fetal bovine serum (FBS) in a humid environment at 37℃ with 5% CO_2_.

### Construction of DDP-resistant cell lines

DDP-resistant LC cells (TU686-R, TU177-R) were derived from TU686 and TU177 cells treated with continuous DDP. In brief, as previously reported [34], DDP-resistant LC cells (TU686-R, TU177-R) were established by progressively exposing TU686 and TU177 cells to increasing concentrations of DDP for over 6 months. Drug-resistant cells were cultured in DMEM with 1 µg/mL DDP to maintain the drug resistance.

### Cell treatment

Specific small interfering RNAs (siRNAs) targeting RBM15, IGF2BP3, FER1L4, or MYC (si-RBM15, si-IGF2BP3, si-FER1L4, si-MYC; Table 1) and a scrambled negative control (si-NC) were obtained from Gene-Pharma (Shanghai, China). The overexpression vectors pcDNA3.1 carrying KDM5B, FER1L4, or KCNQ1OT1 (oe-KDM5B, oe-FER1L4, oe-KCNQ1OT1) were constructed, and a recombinant negative control pcDNA3.1 (oe-NC) was purchased from Gene-Pharma. Cells were cultured for 24 h prior to transfection. Then, according to the manufacturer’s instructions, the corresponding siRNAs or pcDNA3.1 vectors were transiently transfected into drug-resistant cells using Lipofectamine 2000 transfection reagent (Invitrogen, Carlsbad, CA, USA). Transfected cells were collected for further analysis after 48 h. The ferroptosis inhibitor ferrostatin-1 (Fer-1; 2 µM; MedChemExpress, Shanghai, China) was used to treat cells, with dimethyl sulfoxide (DMSO) treatment as a negative control.

**Table 1:** siRNA sequences

siRNAs	Sequences
si-RBM15-1	SS Sequence: GCCUGUUUCAUGAGUUCAAAC
	AS Sequence: UUGAACUCAUGAAACAGGCCG
si-RBM15-2	SS Sequence: GAGGAUGAUCAGCGAGCUAAC
	AS Sequence: UAGCUCGCUGAUCAUCCUCGG
si-RBM15-3	SS Sequence: GGACAAGUCCAGCAGUCGAGG
	AS Sequence: UCGACUGCUGGACUUGUCCAG
si-IGF2BP3-1	SS Sequence: GGUACUAGCUAAGAAAUAAUU
	AS Sequence: UUAUUUCUUAGCUAGUACCAG
si-IGF2BP3-2	SS Sequence: CAGUUAUUAGUUAAAUCAAAU
	AS Sequence: UUGAUUUAACUAAUAACUGUG
si-MYC-1	SS Sequence: GGAUGCUAUUGCUGUUCUAAU
	AS Sequence: UAGAACAGCAAUAGCAUCCUU
si-MYC-2	SS Sequence: GAAUUUCAAUCCUAGUAUAUA
	AS Sequence: UAUACUAGGAUUGAAAUUCUG
si-FER1L4-1	SS Sequence: GGAGAGAUGUCGAGUGACAUC
	AS Sequence: UGUCACUCGACAUCUCUCCGG
si-FER1L4-2	SS Sequence: GGUCAGAGUGUAUGUUGUAAA
	AS Sequence: UACAACAUACACUCUGACCAG
si-FER1L4-3	SS Sequence: GCAAGGACUGCGACUUCUACG
	AS Sequence: UAGAAGUCGCAGUCCUUGCUU

### Cell counting kit-8 (CCK-8) assay

A total of 1000 cells were cultured in 96-well plates (100 µL/well) in a complete growth medium. The medium was then replaced with a complete medium containing DDP. After 24 h, the medium was discarded, and cells were gently washed with phosphate-buffered saline (PBS) and added with 10 µL of CCK-8 medium at 37 °C for 2 h. The absorbance was measured at a wavelength of 450 nm. The optical density (OD) values of each well were compared to the negative control well to evaluate the effect of different concentrations of DDP on cell viability. The cell inhibition rate (IR) was calculated using the formula: IR = [1 - (experimental well OD - blank well OD)/(control well OD - blank well OD)] × 100%. A growth inhibition curve was plotted with DDP drug concentration as the x-axis to calculate the IC_50_ of DDP for different cell groups.

### Colony formation assay

The transfected cells were seeded into 6-well plates at 1 × 10^3^ cells per well and incubated in a medium supplemented with 10% FBS for 14 days. Subsequently, cell colonies were fixed using 4% paraformaldehyde and stained with 0.25% crystal violet (Sigma-Aldrich, St. Louis, MO, USA). Following staining, the number of cell colonies containing more than 50 cells was quantified using ImageJ software (NIH, Bethesda, MD, USA).

### **Measurement of iron**,** malondialdehyde (MDA)**,** glutathione (GSH)**,** and ROS**

The iron content in cells and tumor tissues was measured by Iron Assay kits (Colorimetric) (ab83366, Abcam, Cambridge, MA, USA) according to the manufacturer’s instructions. The concentrations of MDA and GSH in cells and tumor tissues were assessed using a lipid oxidation assay kit (S0131S, Beyotime, Shanghai, China) and a glutathione assay kit (S0052, Beyotime).

ROS levels in cells and tumor tissues were assessed using the peroxidase-sensitive fluorescent probe 2,7-Dichlorodihydrofluorescein diacetate (DFCH-DA) following the manufacturer’s protocol (S0033S, Beyotime). In short, cells were treated with 10 µM DCFH-DA for 30 min at 37 °C and observed under fluorescence microscopy, and the outcomes were reported as relative values.

### m6A quantification analysis

m6A quantification was performed using the m6A RNA methylation quantification kit (ab185912, Abcam). According to the manufacturer’s instructions, the corresponding solutions were added to each well for m6A RNA capture. It was ensured that residual wash buffers were thoroughly removed in each washing step. Finally, the signal was detected when the positive control well turned blue. Then, 100 µL of stop solution was added to each well to terminate the enzyme reaction. After that, the reaction mixture turned yellow. The absorbance was read at 450 nm using a microplate reader.

### Methylated RNA immunoprecipitation (MeRIP)

We combined 1–3 µg of total RNA and m6A spike-in control with 300 µL of IP buffer (50 mM Tris-HCl, pH 7.4, 150 mM NaCl, 0.1% NP40, 40 U/µL RNase Inhibitor) containing 2 µg of anti-m6A antibody (ab208577, Abcam). For each sample, 20 µL of Dynabeads™ M-280 immunoglobulin G (IgG) suspension (11202D, Invitrogen) was blocked with freshly prepared 0.5% bovine serum albumin at 4 °C for 2 h, washed thrice with 300 µL of IP buffer, and then mixed with the prepared total RNA-antibody combination. The RNA was subsequently bound to the m6A antibody-coated beads and rotated at 4 °C for 2 h. Enriched RNA was eluted using 200 µL of elution buffer at 50 °C for 1 h to separate the co-precipitated RNA. The eluted RNA was then subjected to reverse-transcription-quantitative polymerase chain reaction (RT-qPCR) analysis as detailed in the subsequent sections.

### Bioinformatics

The RNAInter v4.0 database (http://www.rnainter.org/) [35] was used to predict the binding of FER1L4 with transcription factors MYC, KCNQ1OT1 with MYC, and MYC with GPX4 or ACSL4. The expression of RBM15, KDM5B, and KCNQ1OT1 in head and neck squamous cell carcinoma was predicted from the TCGA link in the UALCAN database (https://ualcan.path.uab.edu/index.html) [36].

### RIP assays

RIP assays were performed using the Magna RIP™ kit (Millipore, Billerica, MA, USA; Sigma-Aldrich) according to the manufacturer’s instructions. Cells were lysed in RIP buffer, and the cell lysates were incubated with antibodies against IGF2BP3 (ab177477, Abcam), MYC (ab32072, Abcam), and IgG (ab172730, Abcam) overnight at 4 °C. The RNA-protein/antibody complexes were then immunoprecipitated using protein A/G magnetic beads. After incubation with Proteinase K buffer (Omega, Shanghai, China), immunoprecipitated RNA was obtained, and the gene expression was analyzed by RT-qPCR.

### RNA stability assay

To examine the effect of RBM15/IGF2BP3 intervention on the stability of KDM5B mRNA in LC cells, 5 µg/mL actinomycin D (Sigma-Aldrich) was added to the cells. The procedure for isolating total RNA for qPCR analysis was detailed in the subsequent section. Ultimately, the mRNA expression levels at the designated time points were computed and standardized to glyceraldehyde-phosphate dehydrogenase (GAPDH).

### Chromatin immunoprecipitation (ChIP)-qPCR

The EZ-Magna ChIP Kit (Millipore) was used for ChIP analysis of the lncRNA FER1L4 promoter. Briefly, the cell lines were cross-linked in 1% formaldehyde solution for 10 min, followed by quenching with glycine. DNA fragments were obtained by sonication. Cell debris was removed by centrifugation (13,000 g at 4 °C). The supernatant was immunoprecipitated with antibodies against KDM5B (ab306579, Abcam), H3K4me3 (ab213224, Abcam), H3K27me3 (ab192985, Abcam), or IgG (ab205718, Abcam). After 24 h, antibody-enriched DNA-protein complexes were precipitated using protein agarose, collected by brief centrifugation, and then subjected to washing, de-crosslinking (overnight at 65 °C), purification, and amplification of DNA fragments. ChIP-qPCR was performed to analyze the precipitated chromatin DNA using RT-qPCR. The upstream primer for the FER1L4 promoter was 5’-TCACAGCTCTCACTCCCTGA-3’, and the downstream primer was 5’-CTGGAGGTCAATTCAGCCCT-3’. The upstream primer for the KCNQ1OT1 promoter was 5’-CTCTCGGACAGGCAGATGAC-3’, and the downstream primer was 5’-GCACAAGTGACACATCCCCT-3’.

### RNA pull-down assay

The RNA pull-down assay was performed using the RNA-Protein pull-down kit (Pierce, Rockford, IL, USA) according to the manufacturer’s instructions. Briefly, biotin-labeled lncRNA FER1L4 or bio-KCNQ1OT1 was incubated with total protein from cell lysates. The complexes formed were bound to streptavidin-coated Dynabeads and then subjected to Western blot analysis to validate the enriched proteins after elusion and recovery.

### Tumor xenograft assay

Male BALB/c nude mice, 6 weeks old, weighing 20–25 g, were purchased from Charles River (Beijing, China). Lentivirus-mediated short hairpin RNA (shRNA) targeting RBM15 (sh-RBM15) and sh-NC (as a control) were synthesized by Genomeditech (Shanghai, China). TU177-R cells infected with sh-RBM15 or sh-NC (1 × 10^6^) were subcutaneously injected into the nude mice. When the tumor volume reached 100 mm^3^, DDP (2 mg/kg) was injected into the peritoneal cavity twice a week. Tumor volumes were measured with calipers every 7 days using the formula: length × width^2^/2. After 28 days, the mice were euthanized, and the excised tumor tissues were collected for subsequent weighing and analysis.

### Immunohistochemistry (IHC)

All samples were fixed in formalin and embedded in paraffin. For IHC staining, the rehydrated sections underwent antigen retrieval, and endogenous peroxidase activity was blocked in 1% H_2_O_2_/PBS solution. After blocking for 1 h, the sections were incubated overnight at 4 °C with rabbit anti-Ki67 antibody (ab16667, Abcam), followed by the addition of a secondary antibody (ab150077, Abcam) and 30 min of incubation at 37 °C. The sections were then developed using the diaminobenzidine (DAB) immunohistochemistry staining kit, followed by counterstaining with hematoxylin for 1 min. After dehydration and sealing, the sections were observed under a microscope.

### RT-qPCR assay

Total RNA was isolated using the TRIzol reagent. According to the manufacturer’s instructions, RNA was reverse transcribed into complementary DNA (cDNA) using the PrimeScript RT Reagent kit (Takara, Dalian, China). The cDNA was amplified using the Fast SYBR Green PCR kit (Thermo Fisher Scientific, Waltham, MA, USA) on the ABI 7500 RT-PCR System (Thermo Fisher Scientific). mRNA expression levels were detected using GAPDH as an internal control. Fold change was determined using the 2^−ΔΔCt^ method [37]. Primers are showen in Table 2.

**Table 2:** PCR primer sequences

Gene	Sequence (5’-3’)
RBM15	F: GCCTTCCCACCTTGTGAGTT
	R: TCAACCAGTTTTGCACGGAC
IGF2BP3	F: ACTCGGTCCCAAAAAGGCAA
	R: TCCCACTGTAAATGAGGCGG
KDM5B	F: ACCCCTTCGCTTTCATCCAC
	R: CAGTCTCTGGATACGTGGCG
FER1L4	F: GGAACACGGAGGATGTGGTT
	R: CTTGTCATGCTCCAACCCCT
KCNQ1OT1	F: CGCTCCCATCTGCACCTTAT
	R: TTCAGCCCACTCTGAACCAC
MYC	F: GGTAGTGGAAAACCAGCCT
	R: CCGAGTCGTAGTCGAGGTCA
GPX4	F: GCAAGGGCATCCTGGGAAAT
	R: CTTGTCGATGAGGAACTGTGG
GAPDH	F: GGTCCCAGCTTAGGTTCATCA
	R: AATCCGTTCACACCGACCTT

### Western blot assay

Total protein was extracted using radioimmunoprecipitation assay buffer (Solarbio Co. Ltd, Beijing, China) containing protease inhibitors. Protein quantification was performed using the bicinchoninic acid protein assay kit (Thermo Fisher Scientific). Proteins were separated by sodium dodecyl sulfate polyacrylamide gel electrophoresis, transferred onto polyvinylidene fluoride membranes (Millipore), and blocked with 5% skim milk. The membranes were then incubated overnight at 4 °C with primary antibodies diluted at 1:1000, followed by incubation with the corresponding secondary antibody IgG (1:2000, ab313650) for 2 h, with GAPDH as an internal reference. Finally, an enhanced chemiluminescence reagent (Millipore) was used for visualization. The primary antibodies were RBM15 (ab96544), KDM5B (ab306579), IGF2BP3 (ab177477), MYC (ab32072), GPX4 (ab125066), ACSL4 (ab205199), and GAPDH (ab313650), all purchased from Abcam.

### Statistical analysis

All data were analyzed and graphed using SPSS 21.0 statistical software (IBM, Armonk, NY, USA) and GraphPad Prism 8.0 software (GraphPad Software Inc., San Diego, CA, USA). First, normality and homogeneity of variance tests were conducted, which verified that the data were in normal distribution and homogeneity of variance. Comparisons between two groups were analyzed by *t*-tests, and comparisons among multiple groups were analyzed by one-way or two-way analysis of variance (ANOVA), followed by Tukey’s multiple comparisons test for post hoc test. *p* < 0.05 was considered statistically significant.

## Results

### RBM15 promoted DDP resistance in LC cells

**Fig. 1:**
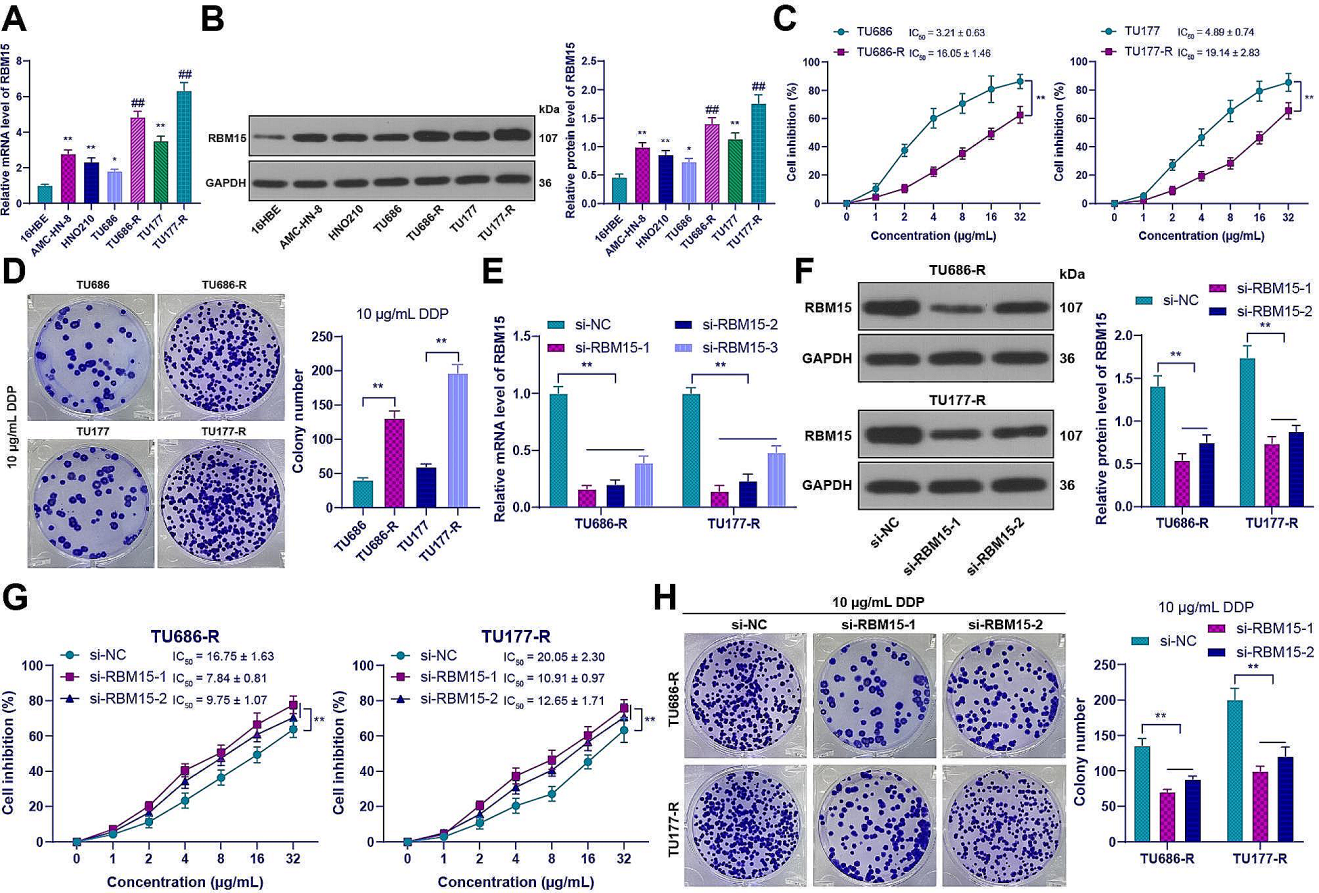
RBM15 promoted DDP resistance in LC cells. (**A**-**B**): The expression of RBM15 in 16HBE cells and LC cancer cells was detected by RT-qPCR and Western blot assay; (**C**): Cell inhibition rate of LC cancer cell lines treated with DDP was measured by CCK-8 assay; (**D**): The number of cell clones after treatment with 10 µg/mL DDP was determined by colony formation assay. si-RBM15 was transfected into drug-resistant cells, with transfection of si-NC as a negative control, and then: (**E**-**F**): The expression of RBM15 in drug-resistant cells was detected by RT-qPCR and Western blot; (**G**): Cell inhibition rate of LC cancer cell lines treated with DDP was measured by CCK-8 assay; (**H**): The number of cell clones after treatment with 10 µg/mL DDP was determined by colony formation assay. The experiments were independently repeated three times, and the data are expressed as mean ± standard deviation. Data in panels A, B, and D: comparisons among multiple groups were analyzed using one-way ANOVA; data in panels C and E-H: comparisons among multiple groups were analyzed using two-way ANOVA. Tukey’s multiple comparisons test was used for post hoc analysis. In panels A-B, * *p* < 0.05 compared to 16HBE cells, ** *p* < 0.01 compared to 16HBE cells, ## *p* < 0.01 compared to the parental cells; in other panels, ** *p* < 0.01

This study aims to investigate the function of RBM15 in DDP resistance in LC. We predicted high expression of RBM15 in head and neck squamous cell carcinoma from the TCGA link in the UALCAN database (https://ualcan.path.uab.edu/index.html) [36] (Supplementary Fig. 1A). Firstly, we examined the expression of RBM15 in LC cells and 16HBE cells. The findings revealed a notable elevation in RBM15 expression in LC cells compared to 16HBE cells (*p* < 0.05, Fig. 1A and B). Subsequently, we selected TU177 and TU686 cells with high and low RBM15 expression levels to establish DDP-resistant cells. Compared to parental cells, DDP-resistant cells exhibited a significant increase in the IC_50_ value for DDP (*p* < 0.01, Fig. 1C), and treatment with 10 µg/mL DDP (selected based on IC_50_) increased cell clone numbers (*p* < 0.01, Fig. 1D). Furthermore, RBM15 expression was further increased in the resistant cell lines compared to the parental cells (*p* < 0.01, Fig. 1A and B). Subsequently, RBM15 expression was downregulated in the resistant cells (*p* < 0.01, Fig. 1E and F), and two siRNAs with good knockdown efficiency were selected for further analysis. The si-RBM15 group showed a decrease in the IC_50_ value for DDP in the resistant cells compared to the si-NC group (*p* < 0.01, Fig. 1G), and treatment with 10 µg/mL DDP resulted in a decrease in cell clone numbers (*p* < 0.01, Fig. 1H). These results indicated that RBM15 was increased in LC cells and promoted DDP resistance.

### RBM15 promoted DDP resistance in LC cells through inhibition of ferroptosis

**Fig. 2:**
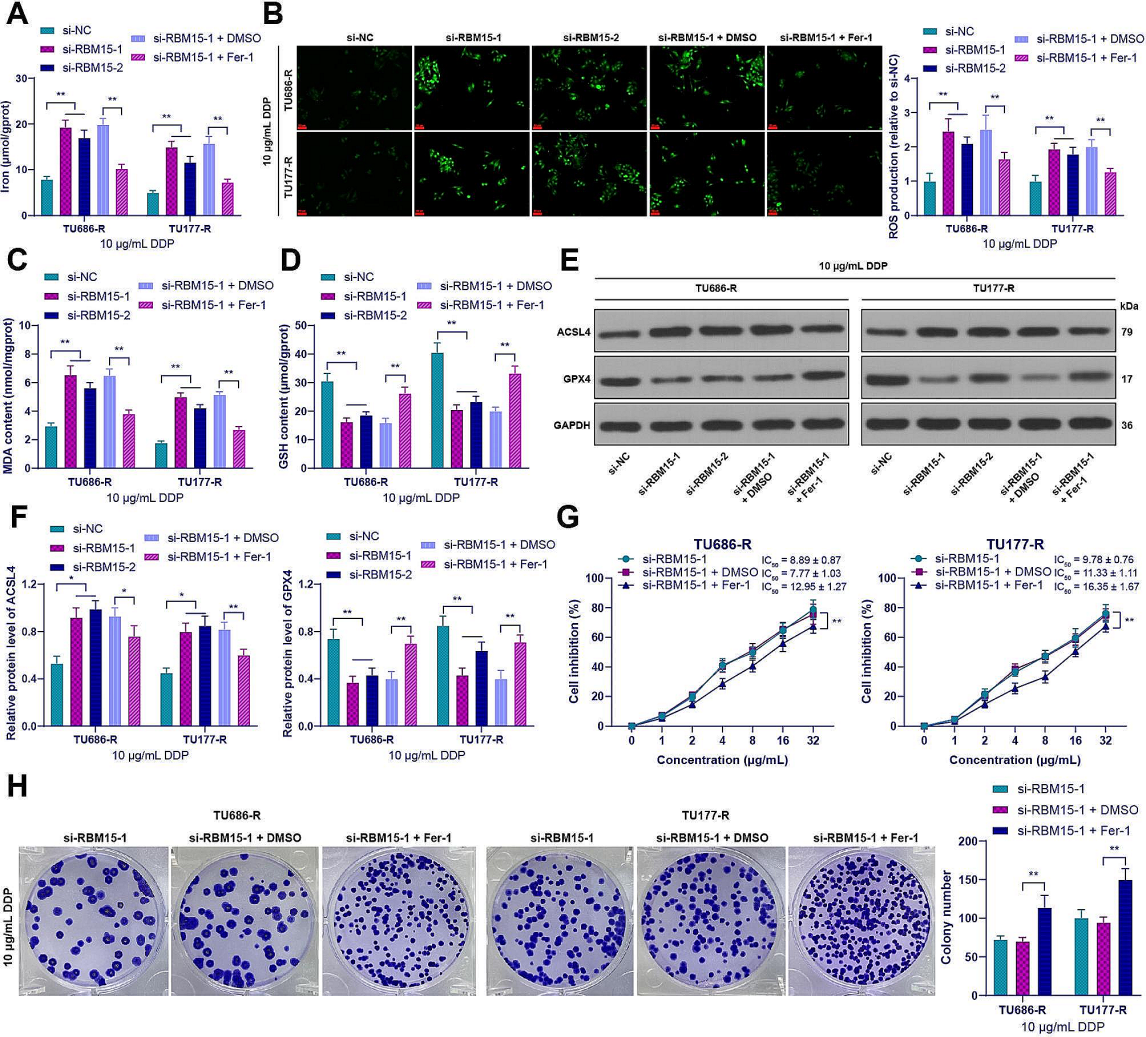
RBM15 promoted DDP resistance in LC cells through inhibition of ferroptosis. After treatment of drug-resistant cells with 10 µg/mL DDP, (**A**): Iron content in cells was measured by colorimetric assay; (**B**): Intracellular ROS levels were measured using the DCFH-DA probe; (**C**): MDA levels in cells were assessed using a lipid peroxidation assay kit; (**D**): GSH content in cells was evaluated using a GSH assay kit; (**E**-**F**): The expression of ACSL4 and GPX4 in cells was detected by Western blot. Drug-resistant cells were treated with the ferroptosis inhibitor Fer-1, with DMSO treatment as a negative control, and then (**G**): Cell inhibition rate of LC cancer cell lines treated with DDP was measured by CCK-8 assay; (**H**): The number of cell clones was measured by colony formation assay. The experiments were independently repeated three times, and the data are expressed as mean ± standard deviation. Data comparisons among multiple groups were analyzed using two-way ANOVA, followed by Tukey’s multiple comparisons test. * *p* < 0.05, ** *p* < 0.01

The role of ferroptosis in cancer chemoresistance has been increasingly elucidated [10, 38]. Subsequently, we examined the alterations of ferroptosis in RBM15-regulated DDP-resistant LC cells. Following RBM15 knockdown, the resistant cells treated with 10 µg/mL of DDP exhibited elevated levels of iron ions, ROS, and MDA, and a reduction in GSH levels (*p* < 0.01, Fig. 2A-D). Compared to si-NC treatment, si-RBM15 treatment resulted in upregulation of ACSL4 expression and downregulation of GPX4 expression in the resistant cells (*p* < 0.05, Fig. 2E and F), indicating that RBM15 knockdown promoted ferroptosis in LC cells. Consequently, we added the ferroptosis inhibitor Fer-1 to the resistant cells, which successfully inhibited the level of ferroptosis in LC cells (*p* < 0.01, Fig. 2A-F). As a result, DDP-resistant cells exhibited a significant increase in the IC_50_ value for DDP (*p* < 0.01, Fig. 2G), and treatment with 10 µg/mL DDP increased in cell clone numbers (*p* < 0.01, Fig. 2H). In summary, RBM15 promoted DDP resistance in LC cells through the inhibition of ferroptosis.

### RBM15 promoted KDM5B by stabilizing KDM5B mRNA levels through m6A modification in an IGF2BP3-dependent manner

**Fig. 3:**
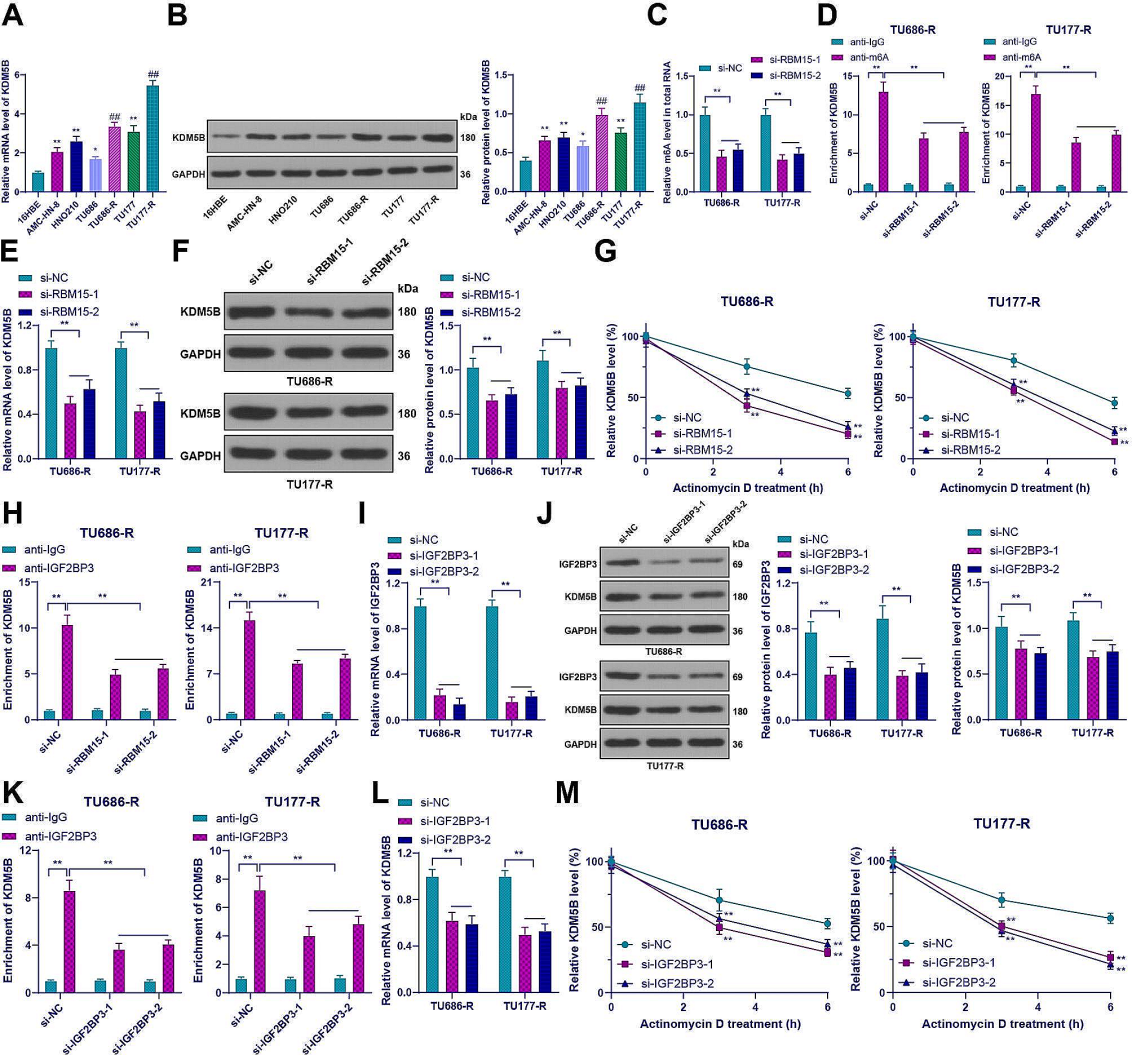
RBM15 promoted KDM5B by stabilizing KDM5B mRNA levels through m6A modification in an IGF2BP3-dependent manner. (**A**-**B**): The expression of KDM5B in 16HBE cells and LC cells was detected by RT-qPCR and Western blot; (**C**): m6A content in cells was measured by colorimetric assay; (**D**): m6A modification of KDM5B was detected by MeRIP-qPCR; (**E**-**F**): The expression of KDM5B in drug-resistant cells with low RBM15 expression was detected by RT-qPCR and Western blot; (**G**): KDM5B mRNA stability was analyzed by qPCR after treatment with actinomycin D; (**H**): The enrichment of IGF2BP3 at the m6A modification sites of KDM5B was assessed by RIP analysis; (**I**): The expression of IGF2BP3 in drug-resistant cells was detected by RT-qPCR after transfection with si-IGF2BP3; (**J**): The expression of IGF2BP3 and KDM5B in drug-resistant cells was detected by Western blot; (**K**): The enrichment of IGF2BP3 at the m6A modification sites of KDM5B was assessed by RIP analysis; (**L**): The expression of KDM5B in drug-resistant cells was detected by RT-qPCR; (**M**): KDM5B mRNA stability was analyzed by qPCR after treatment with actinomycin D. The experiments were independently repeated three times, and the data are expressed as mean ± standard deviation. Data in panels A-B: comparisons among multiple groups were analyzed using one-way ANOVA; data in panels C-M: comparisons among multiple groups were analyzed using two-way ANOVA. Tukey’s multiple comparisons test was used for post hoc analysis. In panels A-B, * *p* < 0.05 compared to 16HBE cells, ** *p* < 0.01 compared to 16HBE cells, ## *p* < 0.01 compared to the parental cells; in panels G and M, ** *p* < 0.01 compared to si-NC; in other panels, * *p* < 0.05, ** *p* < 0.01

RBM15 regulates the stability of TMBIM6 in an IGF2BP3-dependent manner, promoting the progression of laryngeal squamous cell carcinoma [14]. m6A modification has been observed in KDM5B [18, 19], and KDM5B is highly expressed in LC [23, 24]. The prediction of TCGA link in the UALCAN database showed that KDM5B is highly expressed in head and neck squamous cell carcinoma (Supplementary Fig. 1B). Consistent with these findings, our results showed elevated expression of KDM5B in LC, which was further increased in drug-resistant cells (*p* < 0.05, Fig. 3A and B). Hence, we hypothesized that KDM5B might be a potential downstream target of RBM15. Downregulation of RBM15 led to a decrease in m6A levels in drug-resistant LC cells (*p* < 0.01, Fig. 3C). MeRIP-qPCR assay further investigated the m6A modification of KDM5B and the results indicated that the KDM5B mRNA sequence was enriched with m6A modifications, but downregulation of RBM15 resulted in a decrease in m6A modification of KDM5B mRNA (*p* < 0.01, Fig. 3D). Additionally, we observed a decrease in KDM5B expression with downregulation of RBM15 (*p* < 0.01, Fig. 3E and F), and downregulation of RBM15 reduced the stability of KDM5B mRNA in drug-resistant cells (*p* < 0.01, Fig. 3G). These results provide strong evidence for the regulatory role of RBM15 in KDM5B expression.

Next, we investigated whether IGF2BP3 enhanced the stability of KDM5B in an m6A-dependent manner. First, RIP assay showed that IGF2BP3 was enriched at the m6A modification sites of KDM5B, but IGF2BP3 enrichment was decreased after downregulation of RBM15 (*p* < 0.01, Fig. 3H). Subsequently, IGF2BP3 knockdown in drug-resistant cells (*p* < 0.01, Fig. 3I and J) showed decreased enrichment of IGF2BP3 on the m6A modification sites of KDM5B (*p* < 0.01, Fig. 3K). Downregulation of IGF2BP3 decreased KDM5B expression (*p* < 0.01, Fig. 3J and L) and reduced the stability of KDM5B mRNA in drug-resistant cells (*p* < 0.01, Fig. 3M). In summary, RBM15 promoted KDM5B by stabilizing KDM5B mRNA levels through m6A modification in an IGF2BP3-dependent manner.

### Overexpression of KDM5B attenuated the inhibitory effect of RBM15 knockdown on DDP resistance in LC cells by inhibiting ferroptosis

**Fig. 4:**
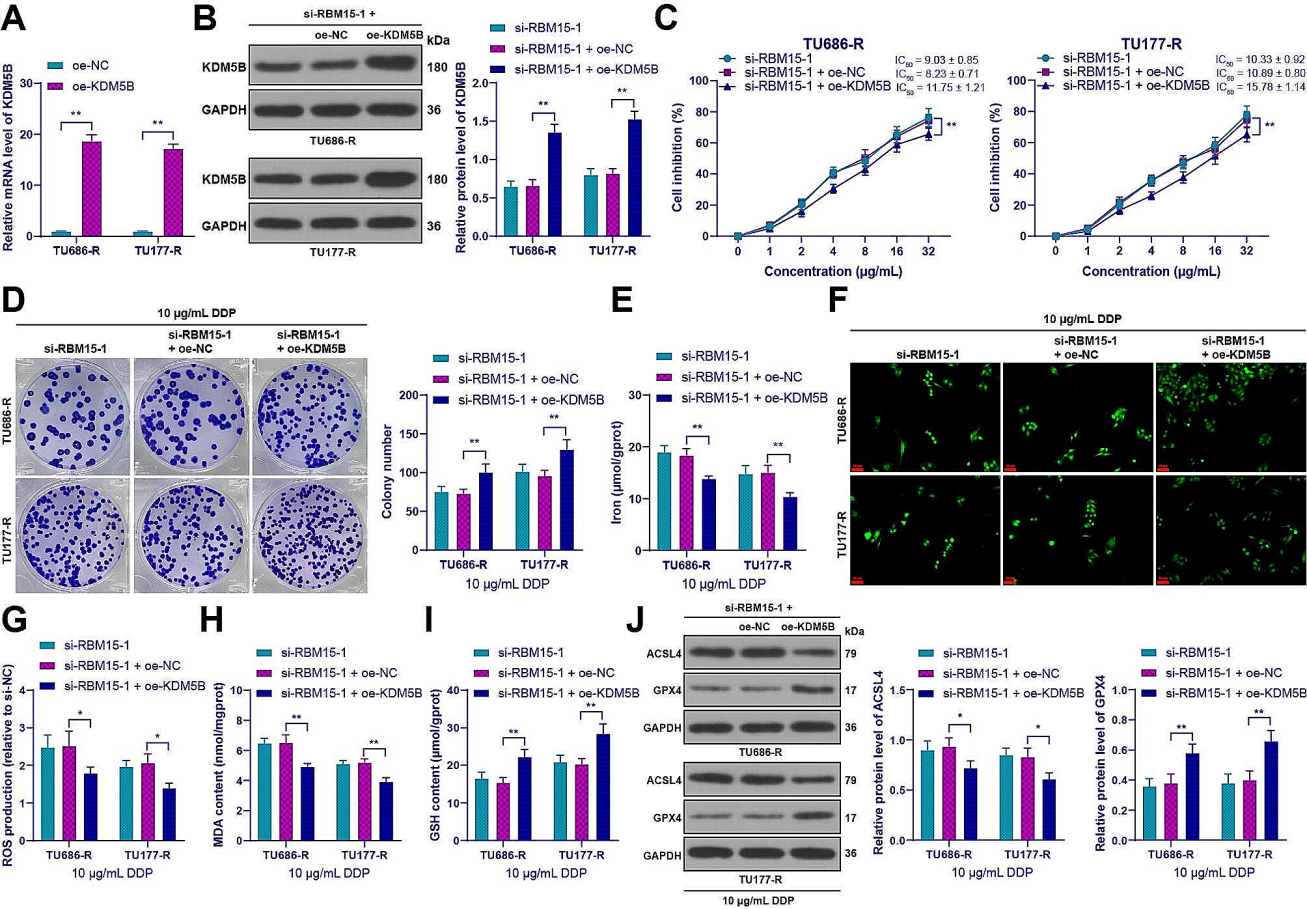
Overexpression of KDM5B attenuated the inhibitory effect of RBM15 knockdown on DDP resistance in LC cells by inhibiting ferroptosis. oe-KDM5B was transfected into drug-resistant cells, with transfection of oe-NC as a negative control. (**A**-**B**): The expression of KDM5B in cells was detected by RT-qPCR and Western blot; (**C**): Cell inhibition rate of LC cancer cell lines treated with DDP was measured by CCK-8 assay. The cells were treated with 10 µg/mL DDP, and then (**D**): The number of cell clones was measured by colony formation assay; (**E**): Iron content in cells was measured by colorimetric assay; (**F**-**G**): Intracellular ROS levels were measured using the DCFH-DA probe; (**H**): MDA levels in cells were assessed using a lipid peroxidation assay kit; (**I**): GSH content in cells was evaluated using a GSH assay kit; (**J**): The expression of ACSL4 and GPX4 in cells was detected by Western blot. The experiments were independently repeated three times, and the data are expressed as mean ± standard deviation. Data comparisons among multiple groups were analyzed using two-way ANOVA, followed by Tukey’s multiple comparisons test. * *p* < 0.05, ** *p* < 0.01

Subsequently, we upregulated the expression of KDM5B in drug-resistant cells (*p* < 0.01, Fig. 4A-B) and conducted combined experiments with si-RBM15-1. Compared to the knockdown of RBM15 alone, the drug-resistant cells in the combined group showed an increase in IC_50_ for DDP (*p* < 0.01, Fig. 4C) and an increase in cell clone number (*p* < 0.01, Fig. 4D). After overexpression of KDM5B, the iron ion content was decreased, and the levels of ROS and MDA were decreased, while the GSH content was increased in drug-resistant cells (*p* < 0.05, Fig. 4E-I). Compared to oe-NC treatment, oe-KDM5B treatment resulted in a decrease in the expression of ACSL4 and an increase in the expression of GPX4 in drug-resistant cells (*p* < 0.05, Fig. 4J). These results suggest that overexpression of KDM5B abated the inhibitory effect of RBM15 knockdown on DDP resistance in LC cells by inhibiting ferroptosis.

### KDM5B regulated the demethylation of the H3K4me3 and H3K27me3 promoters to regulate the expression of FER1L4/KCNQ1OT1

**Fig. 5:**
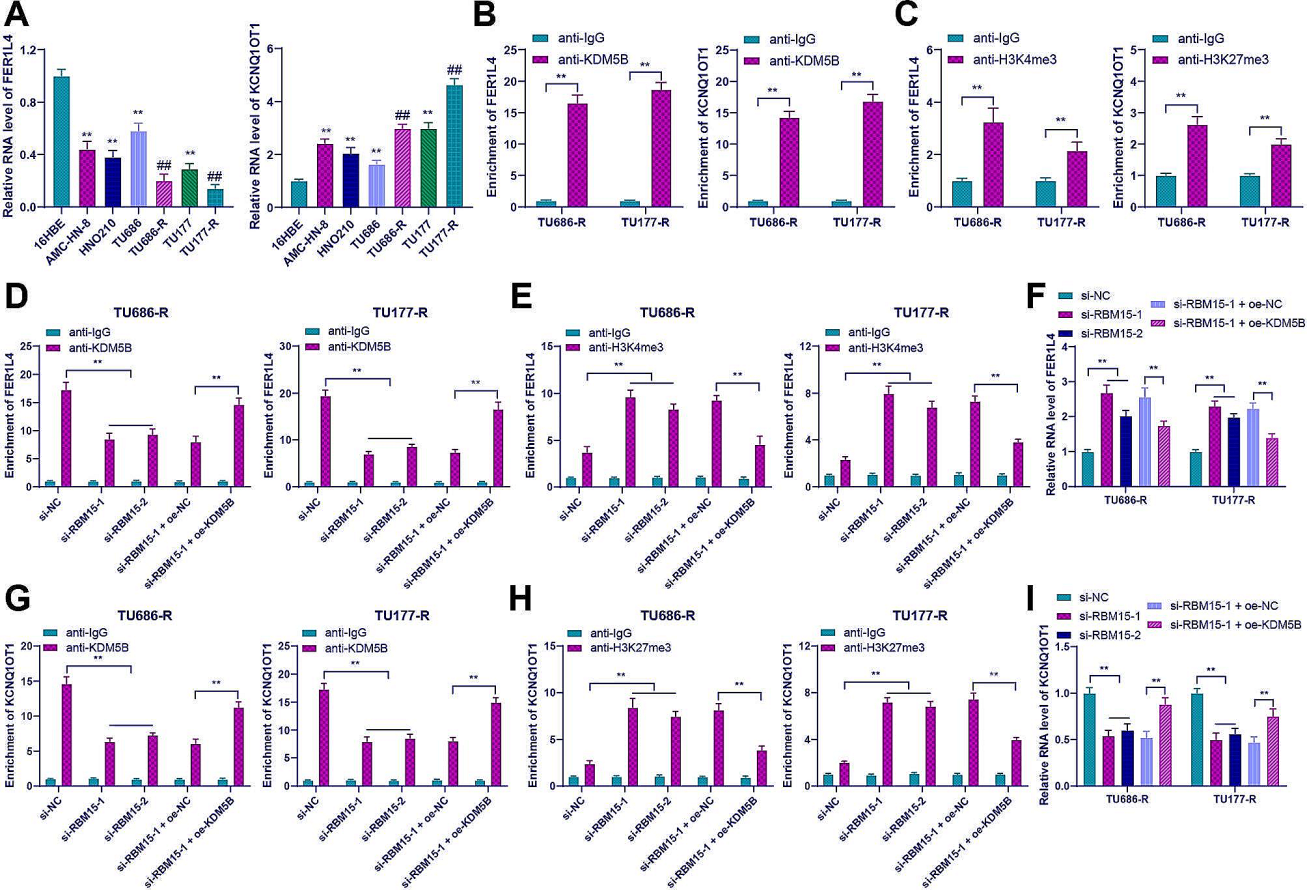
KDM5B regulated the demethylation of the H3K4me3 and H3K27me3 promoters to regulate the expression of FER1L4/KCNQ1OT1. (**A**): The expression of FER1L4 and KCNQ1OT1 in 16HBE cells and LC cells was detected by RT-qPCR; (**B**-**C**): The enrichment of KDM5B, H3K4me3, or H3K27me3 on the FER1L4 or KCNQ1OT1 promoters in drug-resistant cells was assessed by ChIP-qPCR; (**D**-**E**): The enrichment of KDM5B and H3K4me3 on the FER1L4 promoter was analyzed by ChIP-qPCR; (**F**): The expression of FER1L4 in drug-resistant cells from different groups was detected by RT-qPCR; (**G**-**H**): The enrichment of KDM5B and H3K27me3 on the KCNQ1OT1 promoter was assessed by ChIP-qPCR; (**I**): The expression of KCNQ1OT1 in drug-resistant cells from different groups was detected by RT-qPCR. The experiments were independently repeated three times, and the data are expressed as mean ± standard deviation. Data in panel A: comparisons among multiple groups were analyzed using one-way ANOVA; data in panels B-I: comparisons among multiple groups were analyzed using two-way ANOVA. Tukey’s multiple comparisons test was used for post hoc analysis. In panel A, ** *p* < 0.01 compared to 16HBE cells, ## *p* < 0.01 compared to the parental cells; in other panels, ** *p* < 0.01

KDM5B has been shown to regulate the demethylation of H3K4me3 [25], while lncRNA FER1L4 is downregulated in LC [31]. It is hypothesized that KDM5B inhibited the expression of FER1L4 through demethylation of H3K4me3. Our results showed a decrease in FER1L4 expression in LC cells, which was further decreased in drug-resistant cells (*p* < 0.01, Fig. 5A). ChIP results demonstrated the enrichment of both KDM5B and H3K4me3 at the FER1L4 promoter (*p* < 0.01, Fig. 5B and C). However, after RBM15 knockdown, the enrichment of KDM5B on the FER1L4 promoter was decreased, while the enrichment of H3K4me3 was increased. Conversely, overexpression of KDM5B showed the opposite trend (*p* < 0.01, Fig. 5D and E). Meanwhile, RBM15 knockdown upregulated the expression of FER1L4, but overexpression of KDM5B suppressed the upregulation of FER1L4 (*p* < 0.01, Fig. 5F). These results indicated that KDM5B induced demethylation of H3K4me3 on the FER1L4 promoter, thereby inhibiting FER1L4 expression.

KDM5B regulates the demethylation of H3K27me3 [24], while lncRNA KCNQ1OT1 is highly expressed in LC [39]. The prediction of TCGA link in the UALCAN database showed that lncRNA KCNQ1OT1 is highly expressed in head and neck squamous cell carcinoma. We hypothesized that KDM5B promoted KCNQ1OT1 expression through H3K27me3 demethylation. Our results showed an increase in KCNQ1OT1 expression in LC cells, which was further increased in drug-resistant cells (*p* < 0.01, Fig. 5A). ChIP results demonstrated the enrichment of both KDM5B and H3K27me3 at the KCNQ1OT1 promoter (*p* < 0.01, Fig. 5B and C). However, after RBM15 knockdown, the enrichment of KDM5B on the KCNQ1OT1 promoter was decreased, while the enrichment of H3K27me3 was increased. Conversely, overexpression of KDM5B showed the opposite trend (*p* < 0.01, Fig. 5G and H). Similarly, RBM15 knockdown downregulated the expression of KCNQ1OT1, but overexpression of KDM5B suppressed KCNQ1OT1 downregulation (*p* < 0.01, Fig. 5I). These results indicated that KDM5B induced demethylation of H3K27me3 on the KCNQ1OT1 promoter, thereby promoting KCNQ1OT1 expression.

### FER1L4 inhibited the expression of GPX4 by binding to the transcription factor MYC

**Fig. 6:**
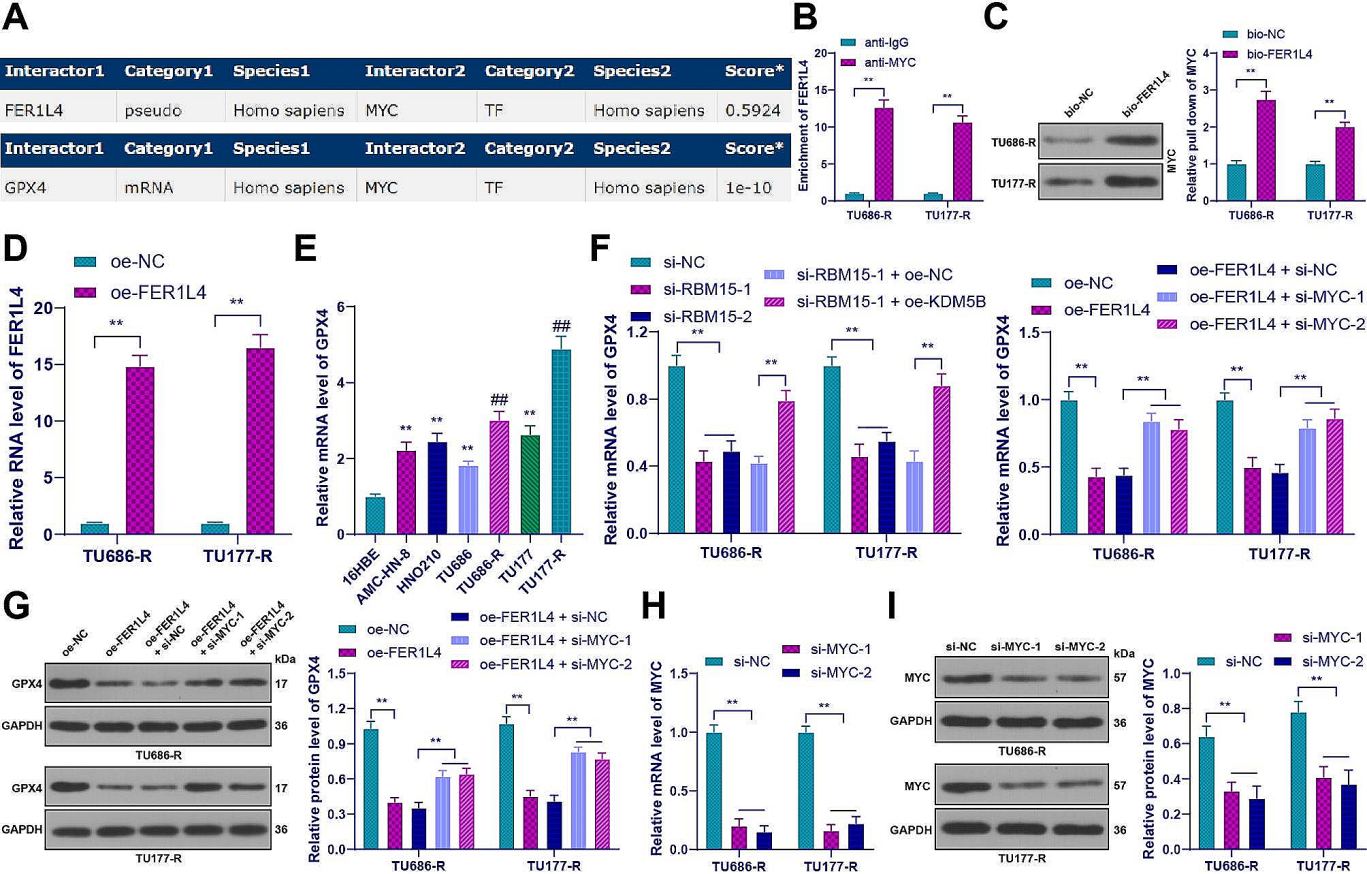
FER1L4 inhibited the expression of GPX4 by binding to the transcription factor MYC. (**A**): The binding between FER1L4, MYC, and GPX4 was predicted using the RNAInter database; (**B**-**C**): The binding of FER1L4 and MYC was analyzed by RIP and RNA pull down assays; (**D**): The expression of FER1L4 in drug-resistant cells transfected with oe-FER1L4 was detected by RT-qPCR; (**E**-**F**): The mRNA levels of GPX4 in 16HBE cells and LC cancer cells were measured by RT-qPCR; (**G**): The expression of GPX4 in drug-resistant cells from different groups was detected by Western blot; (**H**-**I**): After transfection with si-MYC, the expression of MYC in drug-resistant cells from different groups was detected by RT-qPCR and Western blot. The experiments were independently repeated three times, and the data are expressed as mean ± standard deviation. Data in panel E: comparisons among multiple groups were analyzed using one-way ANOVA; data in panels B-D and F-I: comparisons among multiple groups were analyzed using two-way ANOVA. Tukey’s multiple comparisons test was used for post hoc analysis. In panel E, ** *p* < 0.01 compared to 16HBE cells, ## *p* < 0.01 compared to the parental cells; in other panels, ** indicates *p* < 0.01

According to the RNAInter database, it was indicated that FER1L4 could interact with the transcription factor MYC, and MYC in turn can bind to GPX4 (Fig. 6A). This led us to hypothesize that FER1L4 binds to MYC to suppress the expression of GPX4. RIP assay results showed a significant increase in enrichment of FER1L4 in the MYC immunoprecipitation group compared to the IgG group (*p* < 0.01, Fig. 6B). Additionally, RNA pull-down assay showed stronger enrichment of FER1L4 in the bio-FER1L4 group compared to the bio-NC group (*p* < 0.01, Fig. 6C), suggesting a binding relationship between FER1L4 and MYC. To validate the role of MYC in FER1L4-mediated regulation of GPX4, we increased the expression of FER1L4 in drug-resistant cells (*p* < 0.01, Fig. 6D) and found a significant decrease in GPX4 expression (*p* < 0.01, Fig. 6F and G). Conversely, when we decreased the expression of MYC in cells (*p* < 0.01, Fig. 6H and I), the expression of GPX4 was increased (*p* < 0.01, Fig. 6F and G). Additionally, GPX4 mRNA expression was increased in LC cells and further increased in drug-resistant cells. However, after RBM15 knockdown, GPX4 mRNA level was decreased, but KDM5B overexpression increased GPX4 mRNA again (*p* < 0.01, Fig. 6E and F). In conclusion, FER1L4 inhibited the expression of GPX4 by binding to MYC.

### KCNQ1OT1 inhibited the expression of ACSL4 by binding to MYC

**Fig. 7:**
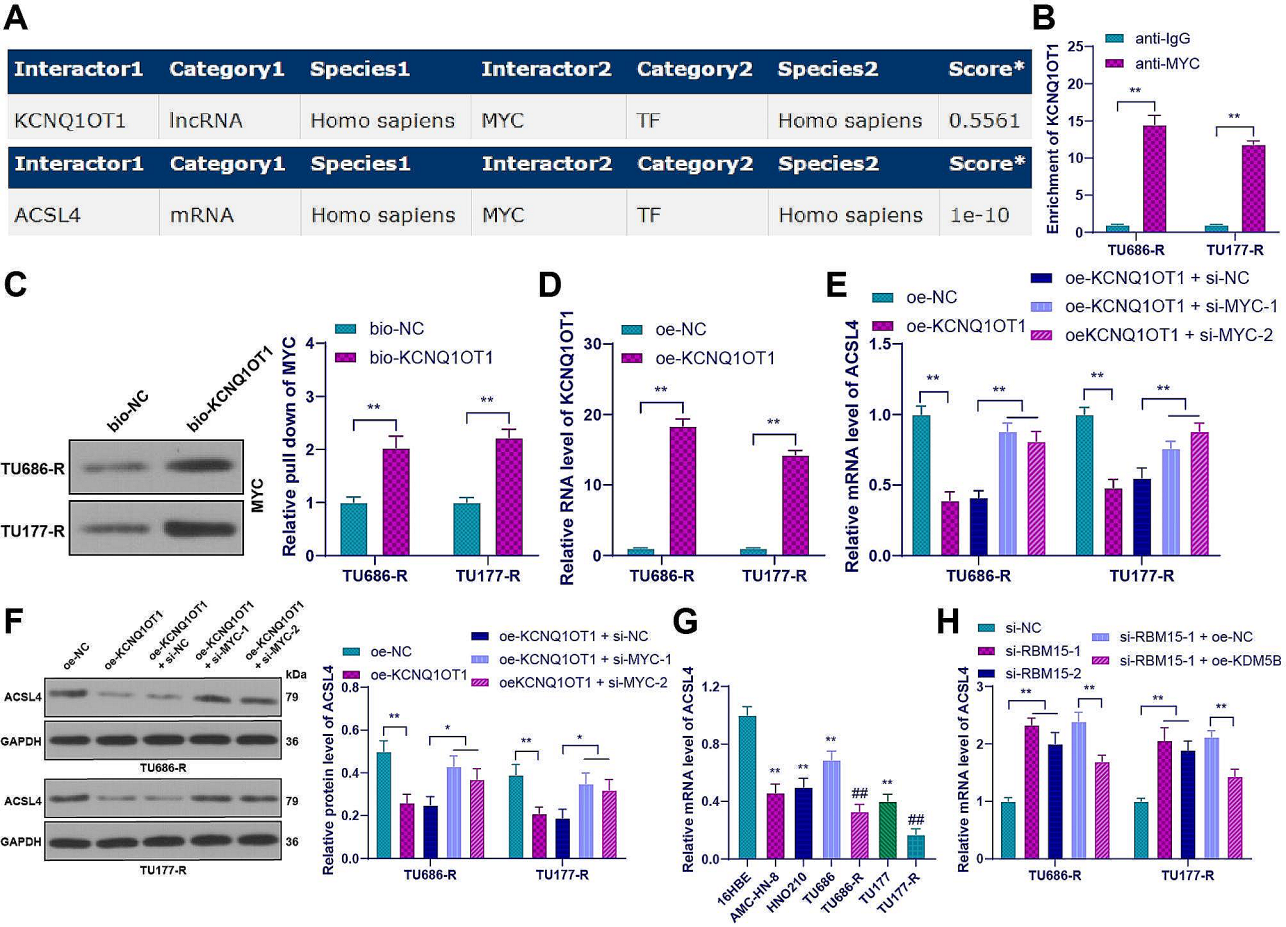
KCNQ1OT1 inhibited the expression of ACSL4 by binding to MYC. (**A**): The binding between KCNQ1OT1, MYC, and ACSL4 was predicted using the RNAInter database; (**B**-**C**): The binding of KCNQ1OT1 and MYC was analyzed by RIP and RNA pull down assays; (**D**): The expression of KCNQ1OT1 in drug-resistant cells transfected with oe-KCNQ1OT1 was detected by RT-qPCR; (**E**-**F**): The expression of ACSL4 in drug-resistant cells from different groups was measured by RT-qPCR and Western blot; (**G**-**H**): The mRNA levels of GPX4 in 16HBE cells and LC cancer cells were detected by RT-qPCR. The experiments were independently repeated three times, and the data are expressed as mean ± standard deviation. Data in panel G: comparisons among multiple groups were analyzed using one-way ANOVA; data in panels B-F, and H: comparisons among multiple groups were analyzed using two-way ANOVA. Tukey’s multiple comparisons test was used for post hoc analysis. In panel G, ** *p* < 0.01 compared to 16HBE cells, ## *p* < 0.01 compared to the parental cells; In other panels, * *p* < 0.05, ** *p* < 0.01

Similarly, the RNAInter database showed that KCNQ1OT1 can bind to MYC, and MYC can bind to ACSL4 (Fig. 7A). Based on this, we hypothesized that KCNQ1OT1 bound to MYC to inhibit the expression of ACSL4. RIP assay showed that the enrichment of KCNQ1OT1 in the MYC group was significantly higher than that in the IgG group (*p* < 0.01, Fig. 7B). RNA pull-down assay showed stronger enrichment of KCNQ1OT1 in the bio-KCNQ1OT1 group compared to the bio-NC group (*p* < 0.01, Fig. 7C), suggesting a binding relationship between KCNQ1OT1 and MYC. To validate the role of MYC in KCNQ1OT1-mediated regulation of ACSL4, we increased the expression of KCNQ1OT1 in drug-resistant cells (*p* < 0.01, Fig. 7D) and found a significant decrease in ACSL4 expression (*p* < 0.01, Fig. 7E and F). Conversely, when we decreased the expression of MYC in LC cells, ACSL4 expression was increased (*p* < 0.05, Fig. 7E and F). Additionally, ACSL4 expression was decreased in LC cells and further decreased in drug-resistant cells. However, after RBM15 knockdown, ACSL4 mRNA level was increased, but overexpression of KDM5B decreased ACSL4 mRNA level again (*p* < 0.01, Fig. 7G and H). In conclusion, KCNQ1OT1 inhibited the expression of ACSL4 by binding to MYC.

### FER1L4 silencing alleviated the inhibitory effect of RBM15 knockdown on DDP resistance in LC cells by inhibiting ferroptosis

**Fig. 8:**
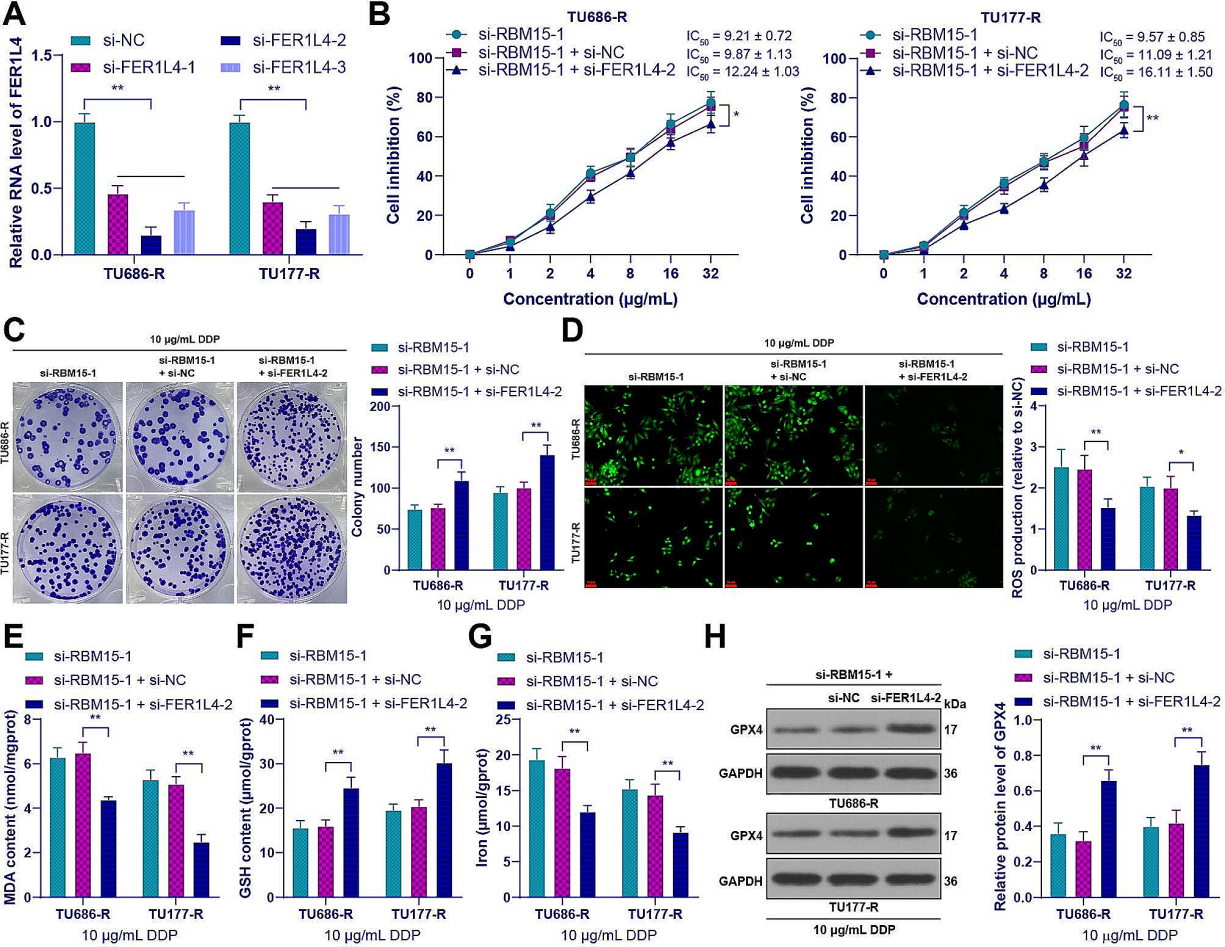
FER1L4 silencing alleviated the inhibitory effect of RBM15 knockdown on DDP resistance in LC cells by inhibiting ferroptosis. si-FER1L4 was tranfected into drug-resistant cells, with transfection of si-NC as a negative control. (**A**): The expression of FER1L4 in cells was detected by RT-qPCR; (**B**): The cell inhibition rate of LC cancer cell lines treated with DDP was measured by CCK-8 assay; The cells were treated with 10 µg/mL DDP, and then (**C**): The number of cell clones was determined by colony formation assay; (**D**): The intracellular ROS levels were measured using the DCFH-DA probe; (**E**): The MDA levels in cells were evaluated using a lipid oxidation assay kit; (**F**): The GSH content in cells was assessed using a GSH assay kit; (**G**): The iron content in cells was measured by colorimetric assay; (**H**): The expression of GPX4 in cells was detected by Western blot. The experiments were independently repeated three times, and the data are expressed as mean ± standard deviation. Data comparisons among multiple groups were performed using two-way ANOVA, followed by Tukey’s multiple comparisons test. * *p* < 0.05, ** *p* < 0.01

To validate the role of FER1L4, we decreased the expression of FER1L4 in drug-resistant cells (*p* < 0.01, Fig. 8A) and conducted combined experiments with si-FER1L4-2 and si-RBM15-1 with better intervention efficiency. Compared to RBM15 knockdown alone, the drug-resistant cells in the combined group showed increases in IC_50_ for DDP (*p* < 0.05, Fig. 8B) and in the number of cell clones (*p* < 0.01, Fig. 8C). After silencing of FER1L4, the levels of ROS and MDA in drug-resistant cells were decreased, the GSH content was increased (*p* < 0.05, Fig. 8D-F), and the level of ferroptosis was decreased (*p* < 0.01, Fig. 8G and H). These results indicated that FER1L4 silencing averted the inhibitory effect of RBM15 knockdown on DDP resistance in LC cells by inhibiting ferroptosis.

### Overexpression of KCNQ1OT1 alleviated the inhibitory effect of RBM15 knockdown on DDP resistance in LC cells by inhibiting ferroptosis

**Fig. 9:**
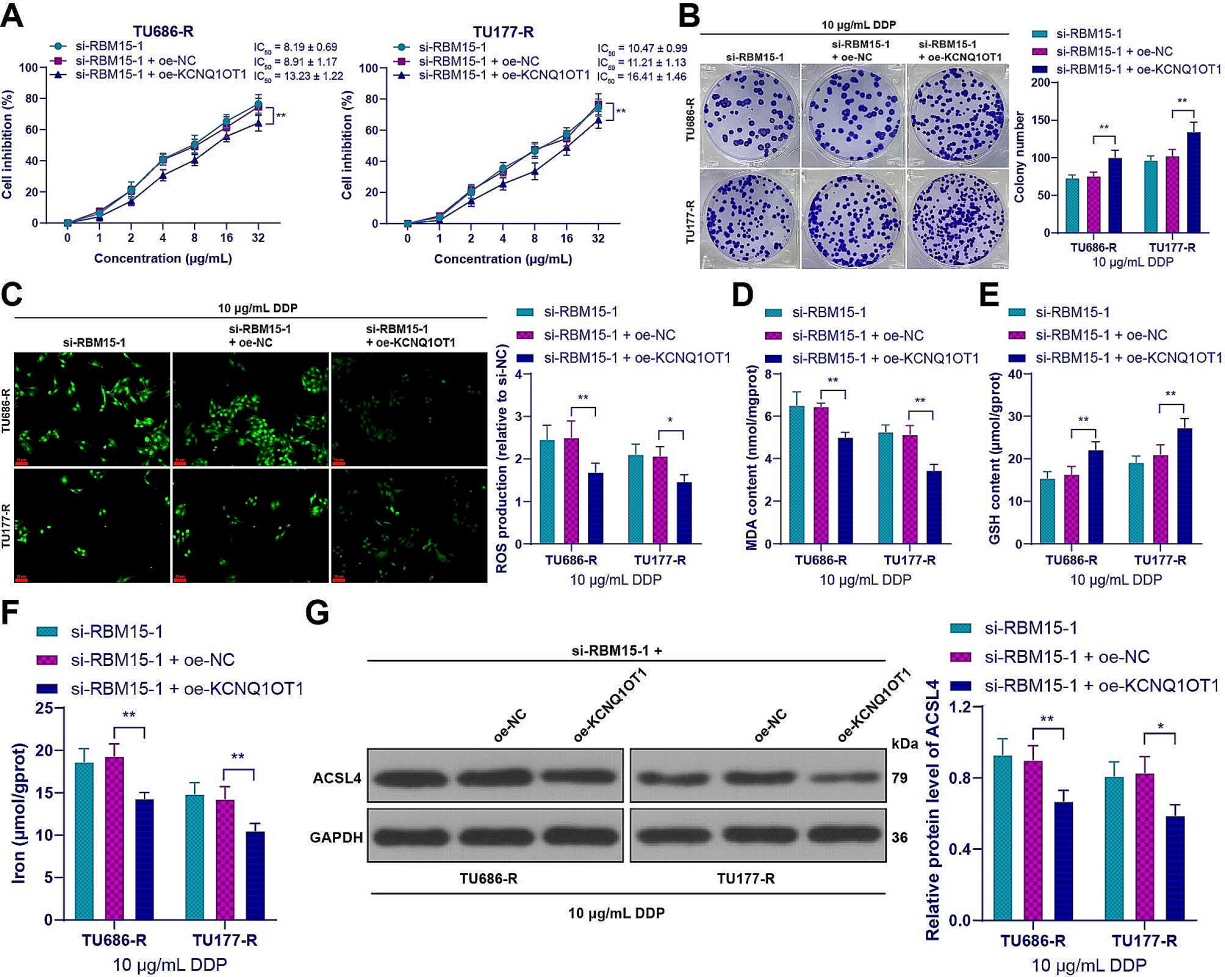
Overexpression of KCNQ1OT1 alleviated the inhibitory effect of RBM15 knockdown on DDP resistance in LC cells by inhibiting iron death. oe-KCNQ1OT1 was transfected into drug-resistant cells, with transfection of oe-NC as a negative control. (**A**): The cell inhibition rate of LC cancer cell lines treated with DDP was measured by CCK-8 assay. The cells were treated with 10 µg/mL DDP, and then (**B**): The number of cell clones was determined by colony formation assay; (**C**): The intracellular ROS levels were measured using the DCFH-DA probe; (**D**): The MDA levels in cells were evaluated using a lipid oxidation assay kit; (**E**): The GSH content in cells was assessed using a GSH assay kit; (**F**): The iron content in cells was measured by colorimetric assay; (**G**): The expression of ACSL4 in cells was detected by Western blot. The experiments were independently repeated three times, and the data are expressed as mean ± standard deviation. Data comparisons among multiple groups were performed using two-way ANOVA, followed by Tukey’s multiple comparisons test. * *p* < 0.05, ** *p* < 0.01

To validate the role of KCNQ1OT1, we increased the expression of KCNQ1OT1 in drug-resistant cells (*p* < 0.01, Fig. 7D) and conducted combined experiments with si-RBM15-1. Compared to RBM15 knockdown alone, the drug-resistant cells in the combined group showed increases in IC_50_ for DDP (*p* < 0.01, Fig. 9A) and in the number of cell clones (*p* < 0.01, Fig. 9B), while the level of ferroptosis was decreased (*p* < 0.05, Fig. 9C-G). These results indicated that overexpression of KCNQ1OT1 nullified the inhibitory effect of RBM15 knockdown on DDP resistance in LC cells by inhibiting ferroptosis.

### RBM15-mediated m6A modification reduces DDP resistance in LC cells by inhibiting ferroptosis

**Fig. 10:**
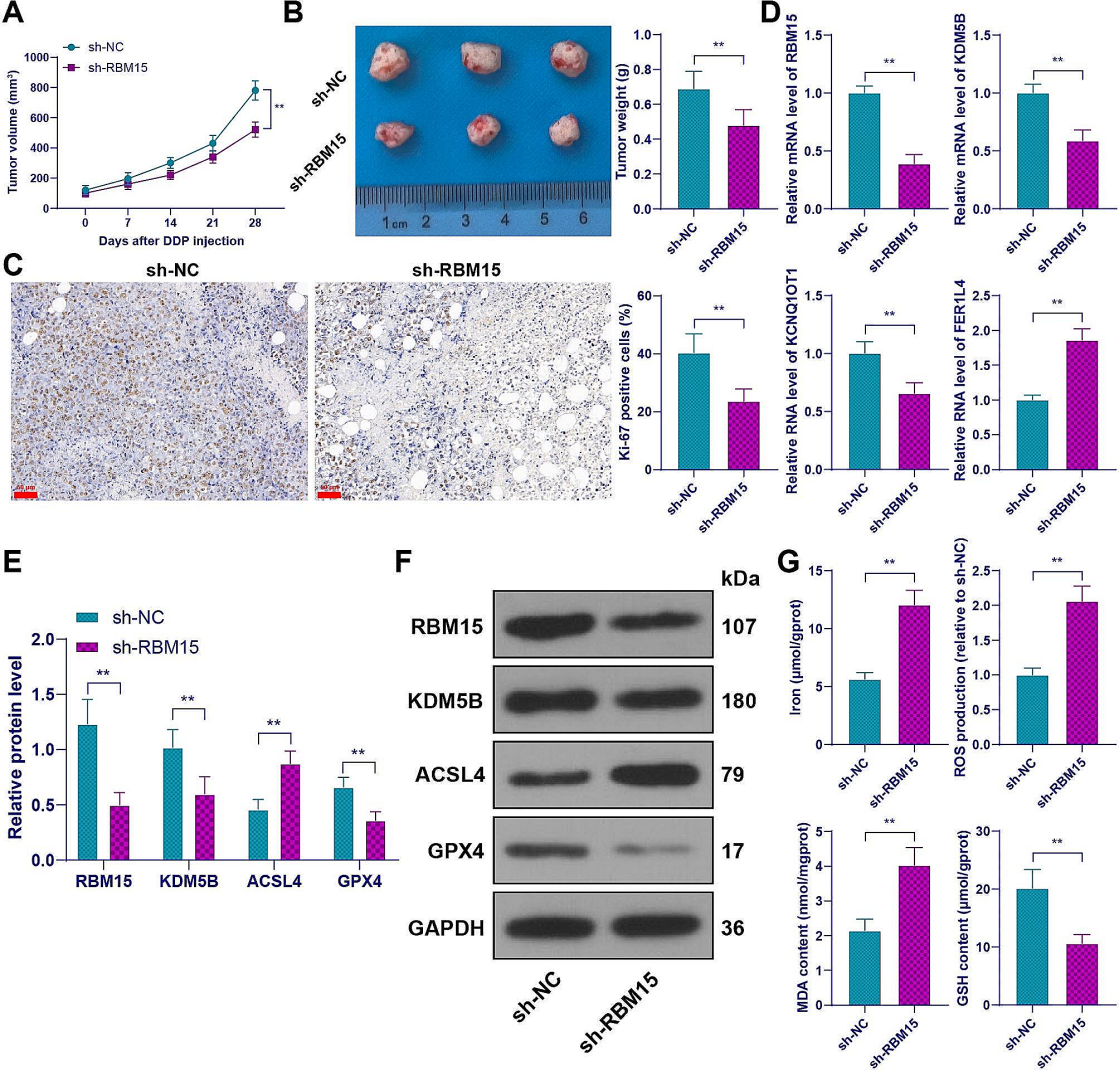
RBM15-mediated m6A modification reduces cisplatin resistance in LC cells by inhibiting ferroptosis. DDP-resistant cells with stably downregulated RBM15 were injected into nude mice. (**A**-**B**): Tumor volume and weight were measured (representative images of the tumors); (**C**): Ki67 positivity rate was detected by IHC; (**D**): Expression of RBM15, KDM5B, KCNQ1OT1, and FER1L4 was assessed by RT-qPCR; (**E**-**F**): Levels of RBM15, KDM5B, ACSL4, and GPX4 were determined by Western blot assay; (**G**): Iron content, ROS level, MDA level, and GSH content in the tissues. *N* = 6. The experiments were independently repeated three times, and the data are expressed as mean ± standard deviation. Data in panels B, C, D, and G: comparison between the two groups was analyzed using *t-*tests; data in panels A and E: comparisons among multiple groups were analyzed using two-way ANOVA, followed by Tukey’s multiple comparisons test. ** *p* < 0.01

Finally, we established a xenograft nude mouse model to validate the mechanism in vivo. Downregulation of RBM15 inhibited tumor growth, as evidenced by reduced tumor volume, decreased weight, and a lower Ki67 positivity rate (*p* < 0.05, Fig. 10A-C). Expression analysis of the excised tumor tissues revealed that compared to the sh-NC group, the sh-RBM15 group exhibited decreased expression of RBM15, KDM5B, and KCNQ1OT1, and increased expression of FER1L4 (*p* < 0.01, Fig. 10D-F). Furthermore, downregulation of RBM15 promoted ferroptosis in the tissues, as indicated by increased levels of iron ions, ROS, MDA, and ACSL4 expression, and decreased levels of GSH and GPX4 expression (*p* < 0.01, Fig. 10F-G). In summary, RBM15-mediated m6A modification reduces DDP resistance in LC cells by inhibiting ferroptosis.

**Fig. 11:**
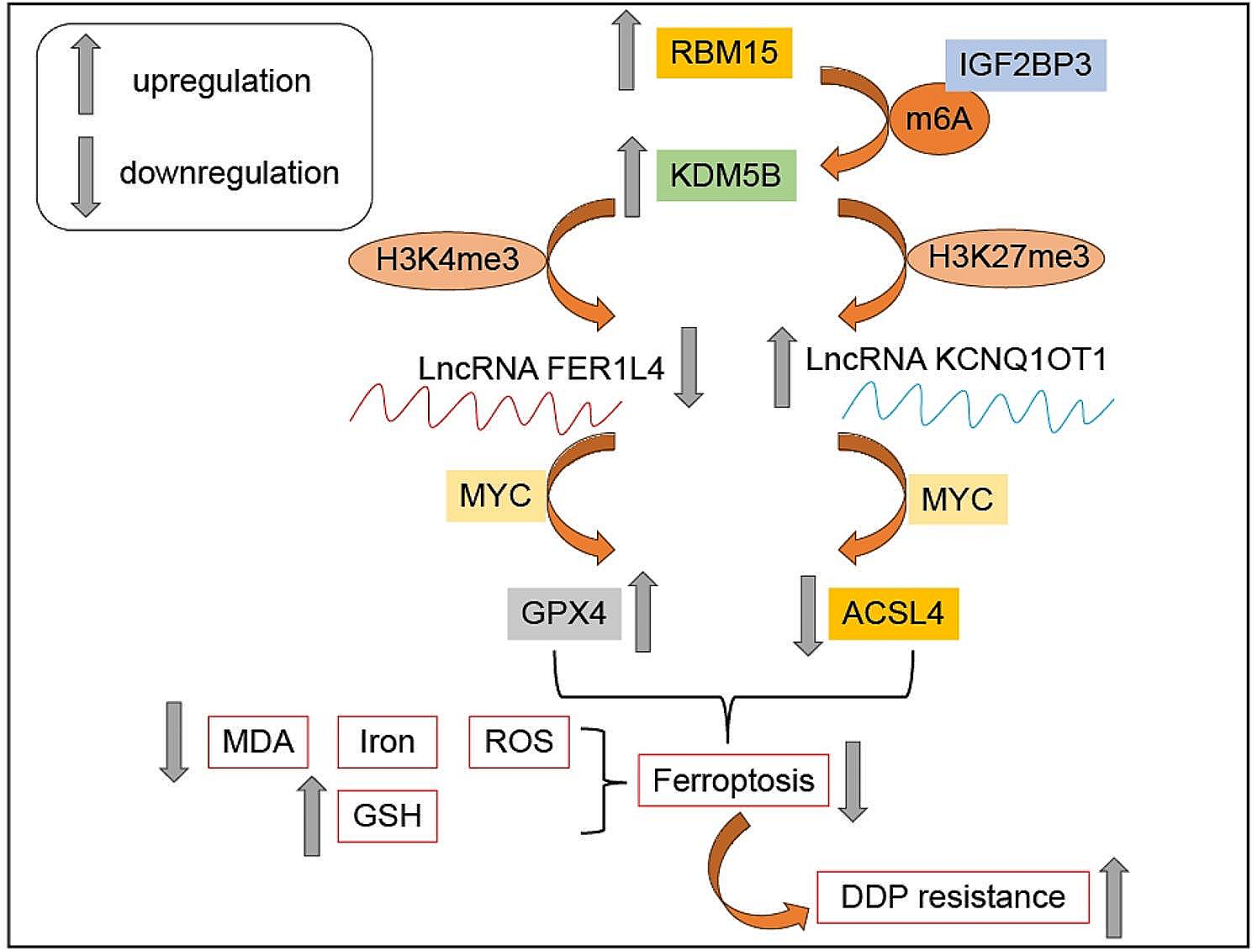
Epigenetic mechanism of RBM15 in affecting cisplatin resistance in laryngeal carcinoma cells by regulating ferroptosis. RBM15 regulated the stability of KDM5B in an IGF2BP3-dependent manner, promoting the expression of KDM5B. KDM5B downregulated FER1L4 through demethylation of H3K4me3 and H3K27me3 while upregulated KCNQ1OT1. FER1L4 and KCNQ1OT1, respectively, bounded to MYC, inhibiting the expression of GPX4 and ACSL4. RBM15 promoted DDP resistance in LC cells by inhibiting ferroptosis

## Discussion

Chemotherapy resistance remains a major challenge in cancer treatment, and understanding the underlying mechanisms is crucial for developing effective therapeutic strategies. In this study, we investigated the role of RBM15 in mediating DDP resistance in LC and the obtained findings demonstrated that RBM15 regulated the stability of KDM5B in an IGF2BP3-dependent manner and promoted KDM5B expression. On one hand, overexpression of KDM5B reduced H3K4me3 levels on the FER1L4 promoter to suppress FER1L4 expression. Consequently, the binding between FER1L4 and MYC was decreased and GPX4 was upregulated. On the other hand, overexpression of KDM5B decreased H3K27me3 levels on the KCNQ1OT1 promoter and promoted KCNQ1OT1 expression. Consequently, the binding between KCNQ1OT1 and MYC was increased and ACSL4 was repressed. Ultimately, the upregulation of GPX4 and downregulation of ACSL4 inhibited ferroptosis and promoted DDP resistance in LC cells (Fig. 11).

Reversing the resistance of cancer to chemotherapy, targeted therapy, and immunotherapy by promoting ferroptosis is an effective way to fight cancer [40]. Importantly, overexpression of RBM15 confers resistance to commonly used chemotherapeutic agents in cancers, including paclitaxel and docetaxel [15, 41]. Our study novelty found that RBM15 was overexpressed in DDP-resistant LC cells. RBM15 silencing-mediated m6A methylation modification can inhibit the growth of colorectal cancer and prevent its liver metastasis [42]. Knockdown of RBM15 significantly increases Fe^2+^ and lipid peroxidation, promotes ferroptosis, and inhibits lung cancer cell growth [43]. Moreover, RBM15 is significantly overexpressed in LC, which leads to increased tumor volume and invasion and is associated with poor prognosis of patients [14]. Consistently, our study demonstrated that RBM15 knockdown reduced the IC_50_ of DDP and improved the effect of DDP treatment in drug-resistant cells. Mechanistically, RBM15 downregulation increased iron ion content, ROS, and MDA levels, and decreased GSH levels in drug-resistant cells. Meanwhile, in LC cells with silencing RBM15, ACSL4 was increased and GPX4 was decreased. These results suggest that RBM15 knockdown repressed DDP resistance in LC cells by inducing ferroptosis in LC cells.

KDM5B is a downstream mechanism of RBM15. Knockdown of KDM5B inhibits the proliferation, migration, and invasion of breast cancer cells [44]. The loss of KDM5B inhibits the proliferation of prostate cancer cells by inhibiting the PI3K/AKT signaling [45]. Notably, serving as an independent prognostic indicator for LC, elevated KDM5B expression may contribute to the proliferation of cancer cells by modulating H3K4me3 levels in LC. Furthermore, KDM5B overexpression is linked to the invasion and unfavorable prognosis of LC cells [23]. Our study found that KDM5B was enriched in m6A modifications and regulated by RBM15 and that KDM5B was decreased with RBM15 depletion. IGF2BP3 has been proposed as an m6A reader and m6A direct binding protein that preferentially recognizes m6A-modified mRNAs and promotes the stabilization and translation of mRNA targets in an m6A-dependent manner [46]. This study identified that IGF2BP3 was enriched at the m6A modification site of KDM5B, and RBM15 stabilized KDM5B mRNA level and promoted KDM5B expression through IGF2BP3-dependent m6A modification. Furthermore, overexpression of KDM5B inhibited ferroptosis in LC cells, thus aggravating the DDP resistance of LC cells. Another study reported that KDM5B knockdown reduced neuroblastoma tumor sphere size and significantly enhanced DDP sensitivity [47]. Although there are currently no reports on KDM5B regulating cancer through ferroptosis, a study has shown that inhibiting KDM1 can reduce GSH production through H3K9me2 modification and enhance lipid peroxidation and ROS accumulation, thus increasing ferroptosis and exhibiting anti-tumor effects on non-small cell lung cancer [48]. We speculate that inhibition of KDM5B may have a similar mechanism for ferroptosis in LC, which needs to be further verified in future experiments.

Next, we found that KDM5B inhibited FER1L4 expression through H3K4me3 demethylation and promoted KCNQ1OT1 expression through H3K27me3 demethylation. FER1L4 functions differently in various tumors. For instance, in oral squamous cell carcinoma, FER1L4 exhibits elevated expression and functions as a carcinogen by targeting the miR-133a-5p/Prx1 axis, thereby enhancing cancer cell viability and diminishing overall patient survival [28]. On the contrary, FER1L4 expression is inhibited in osteosarcoma, and overexpression of FER1L4 can alleviate osteosarcoma cell apoptosis and epithelial-mesenchymal transition by inhibiting the PI3K/AKT pathway [49]. Of note, overexpression of FER1L4 can down-regulate the AKT/ERK pathway, inhibit LC cell viability and proliferation, and promote apoptosis [31]. Our study revealed that FER1L4 is downregulated in LC and knockdown of FER1L4 promoted DDP resistance of LC cells by inhibiting ferroptosis. Elevated FER1L4 can promote the sensitivity of ovarian cancer cells to paclitaxel treatment [50]. However, in liver cancer, overexpression of FER1L4 leads to DDP resistance, suggesting that the specific mechanism of FER1L4 in different cancers still needs to be explored [29].

KCNQ1OT1 typically acts as an oncogenic factor. KCNQ1OT1 upregulation in ovarian cancer contributes to the enhancement of proliferation and migration of ovarian cancer cells by sponging miR-142-5p [51]. Knockdown of KCNQ1OT1 in tongue squamous cell carcinoma inhibited the survival rate, proliferation, migration, invasion, and epithelial-mesenchymal transition of tongue squamous cell carcinoma cells [52]. Importantly, KCNQ1OT1 deletion inhibits LC cell proliferation, migration, and invasion through m6A modification [39]. Moreover, KCNQ1OT1 is up-regulated in DDP-resistant nasopharyngeal carcinoma cells, with the potential to treat patients with DDP-resistant nasopharyngeal carcinoma [53]. Curcumin treatment can effectively down-regulate KCNQ1OT1, thereby reversing the DDP resistance of colorectal cancer cells [54]. Our results showed that overexpression of KCNQ1OT1 alleviated the inhibitory effect of low RBM15 expression on DDP resistance of LC cells by inhibiting ferroptosis. Interestingly, KCNQ1OT1 can increase ACSL4 stability, promote the production of lipid ROS, and activate ferroptosis in normal cells, such as cardiomyocytes and hepatocytes [55, 56].

Furthermore, we validated the downstream mechanism of FER1L4/KCNQ1OT1. FER1L4 inhibited GPX4 expression by binding to MYC and KCNQ1OT1 bound to MYC to repress ACSL4 expression. c-MYC is highly amplified in LC and increases with the severity of laryngeal lesions [57]. c-MYC is significantly upregulated in ovarian cancer and inhibits ferroptosis and ROS in ovarian cancer cells [58]. In neuroblastoma, elevated expression of MYCN, a member of the MYC family, can increase lipid peroxidation and promote ferroptosis, a process that may be associated with the use of GPX4 inhibitors [59]. Inhibition of GPX4 increased iron content in LC cells and promoted ferroptosis [60]. In addition, inhibition of GPX4 can induce ferroptosis in DDP-resistant gastric cancer cells and attenuate DDP-resistance of gastric cancer [61]. Our study found that GPX4 was upregulated in LC cells and inhibited ferroptosis, leading to enhanced DDP resistance. Meanwhile, knockdown of ACSL4 can inhibit cell proliferation by regulating c-MYC in multiple myeloma [62]. Accumulating evidence has confirmed that ACSL4 can enhance the sensitivity of cancer cells to drug treatment by promoting ferroptosis, such as renal cell carcinoma [63] and colorectal cancer [64]. High expression of ACSL4 can inhibit DDP resistance in triple-negative breast cancer by promoting ferroptosis [65]. However, there are few reports on ACSL4 in LC. Our study first found that ACSL4 expression was decreased in both LC cells and further decreased in drug-resistant cells. Overexpression of KCNQ1OT1 or knockdown of MYC decreased ACSL4 expression, leading to ferroptosis inhibition in LC cells.

There are some limitations in this study. First, we only investigated the downstream mechanisms of RBM15 and did not study the upstream mechanisms of RBM15. Second, the association between RBM15 and the therapeutic efficacy of DDP in patients remains to be verified, and the expression of relevant factors in DDP-resistant patients is still not clear. Third, it is still unclear whether MYC directly binds to the GPX4 or ACSL4 promoters. Fourth, we did not explore whether FER1L4 functioned as a competitive endogenous RNA (ceRNA), and the regulatory effects of FER1L4 on ACSL4 and the regulatory effects of KCNQ1OT1 on GPX4 still need to be validated. In the future, we will validate our mechanisms in animal experiments, analyze the therapeutic effect of DDP in LC patients upon RBM15 regulation, investigate whether affecting the activity of RBM15 and KDM5B through drug intervention would impact DDP resistance, verify the regulation of GPX4/ACSL4 by FER1L4/KCNQ1OT1, explore the function of FER1L4 in the ceRNA mechanism and the reasons for the high expression of RBM15 in LC, so as to provide more theoretical knowledge for LC chemotherapy.

## Conclusion

In conclusion, our study clarified that RBM15 affected the expression of GPX4/ACSL4 by the KDM5B/FER1L4/KCNQ1OT1 axis through IGF2BP3-dependent m6A modification, thereby inhibiting ferroptosis and promoting DDP resistance of LC cells.

### Electronic supplementary material

Below is the link to the electronic supplementary material.

Supplementary Material 1

## Data Availability

No datasets were generated or analysed during the current study.
