# Supplementary figures and images for: Epigenetic mechanism of RBM15 in affecting cisplatin resistance in laryngeal carcinoma cells by regulating ferroptosis
Source: Biol Direct. 2024 Jul 23;19:57. doi: 10.1186/s13062-024-00499-6 (PMC11264397; doi:10.1186/s13062-024-00499-6)

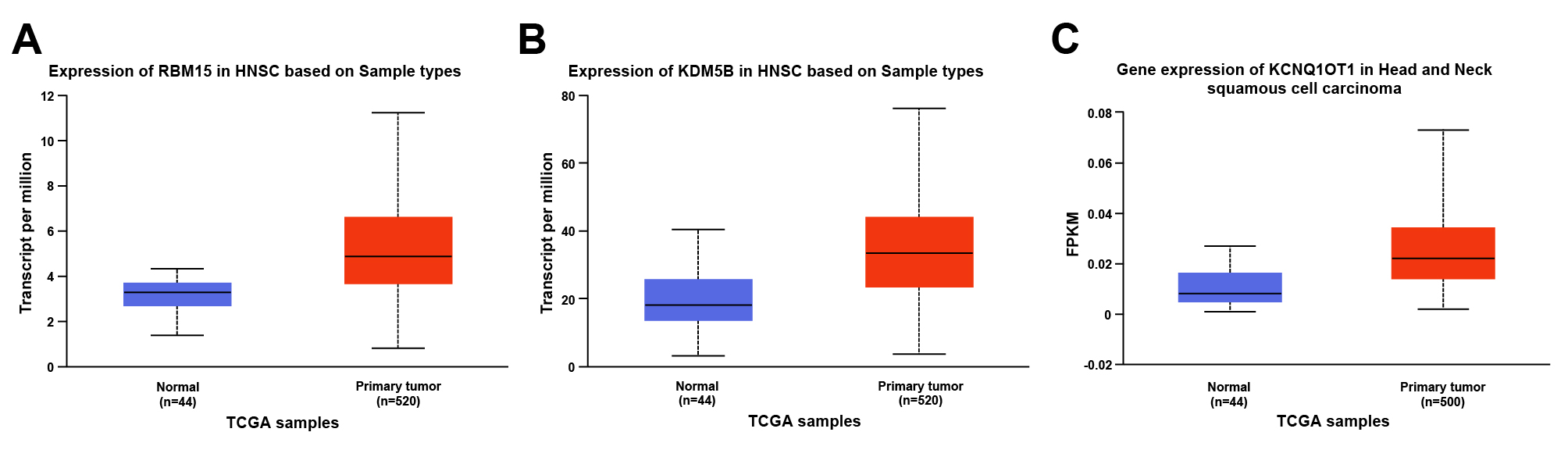

Supplement: Supplementary file 1 — Supplementary Material 1 [file 13062_2024_499_MOESM1_ESM.jpg]
